# Supplementary material for: Conditional chemoconnectomics (cCCTomics) as a strategy for efficient and conditional targeting of chemical transmission
Source: eLife. 2024 Apr 30;12:RP91927. doi: 10.7554/eLife.91927 (PMC11060718; doi:10.7554/eLife.91927)
Supplement: Figure 3—source data 3. [file elife-91927-fig3-data3.zip › Figure 3 source data3 part 2.zip!/Figure 3-Source data 3-part2/1052-13-CRISPResso_Report_ejedac2.zip!/CRISPResso_on_1052_14/1a.Read_barplot.pdf]

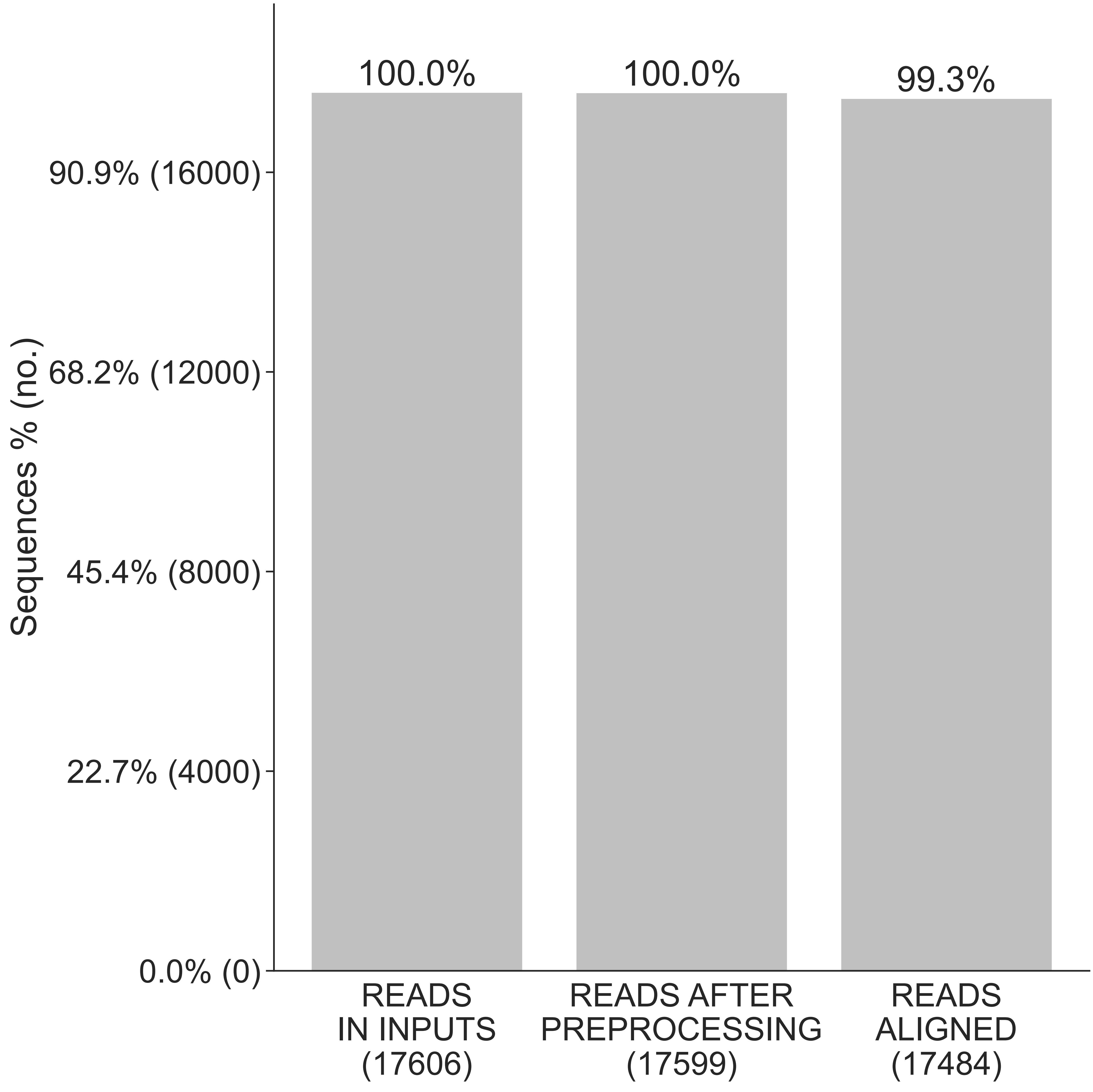
100.0%
100.0%
99.3%
Sequences % (no.)
90.9% (16000)
68.2% (12000)
45.4% (8000)
22.7% (4000)
0.0% (0)
READS IN INPUTS (17606)
READS AFTER PREPROCESSING (17599)
READS ALIGNED (17484)
